# EB-OCT: a potential strategy on early diagnosis and treatment for lung cancer

**DOI:** 10.3389/fonc.2023.1156218

**Published:** 2023-04-25

**Authors:** Hang Long, Jiaqi Ji, Lijuan Chen, Jiayue Feng, Jie Liao, Yang Yang

**Affiliations:** ^1^ Department of Respiratory and Critical Medicine, Sichuan Provincial People’s Hospital, Sichuan Academy of Medical Sciences, Chengdu, Sichuan, China; ^2^ Department of Cardiology, Sichuan Provincial People’s Hospital, Sichuan Academy of Medical Sciences, Chengdu, Sichuan, China

**Keywords:** endobronchial optical coherence tomography, endobronchoscopy, lung cancer, diagnosis, treatment

## Abstract

Lung cancer is the leading cause of cancer-related death in China and the world, mainly attributed to delayed diagnosis, given that currently available early screening strategies exhibit limited value. Endobronchial optical coherence tomography (EB-OCT) has the characteristics of non-invasiveness, accuracy, and repeatability. Importantly, the combination of EB-OCT with existing technologies represents a potential approach for early screening and diagnosis. In this review, we introduce the structure and strengths of EB-OCT. Furthermore, we provide a comprehensive overview of the application of EB-OCT on early screening and diagnosis of lung cancer from *in vivo* experiments to clinical studies, including differential diagnosis of airway lesions, early screening for lung cancer, lung nodules, lymph node biopsy and localization and palliative treatment of lung cancer. Moreover, the bottlenecks and difficulties in developing and popularizing EB-OCT for diagnosis and treatment during clinical practice are analyzed. The characteristics of OCT images of normal and cancerous lung tissues were in good agreement with the results of pathology, which could be used to judge the nature of lung lesions in real time. In addition, EB-OCT can be used as an assistant to biopsy of pulmonary nodules and improve the success rate of biopsy. EB-OCT also plays an auxiliary role in the treatment of lung cancer. In conclusion, EB-OCT is non-invasive, safe and accurate in real-time. It is of great significance in the diagnosis of lung cancer and suitable for clinical application and is expected to become an important diagnostic method for lung cancer in the future.

## 1 Introduction

Current evidence suggests lung cancer is the leading cause of cancer-related death in China and the world, with poor prognosis attributed to delayed diagnosis (1–3). The poor specificity of symptoms accounts for the low diagnosis rates of early-stage lung cancer, while the emergence of chest pain, hemoptysis, and dyspnea often indicate advanced lung cancer. Current evidence suggests that the 5-year survival rate of patients with early or intermediate-stage lung cancer (more than 70%) is significantly higher than late-stage lung cancer (less than 15%) (4). Therefore, early screening, diagnosis and treatment of lung cancer can significantly improve patient survival and prognosis (1, 5–7).

In clinical practice, low-dose computed tomography (LDCT), positron emission tomography/computed tomography (PET/CT) and tumor biomarkers are commonly used to screen solitary pulmonary nodules (SPNs) (4, 7–10). It has been reported that the false positive rate of high-resolution computed tomography (HRCT) is 96.4% (4), which makes it difficult to ascertain the nature of pulmonary lesions. SPNs detection by LDCT requires long-term and multidisciplinary follow-ups leading to cumulative radiation exposure and psychological and economic burdens. In addition, traditional tumor biomarkers and PET/CT are less sensitive and expensive for early diagnosis of lung cancer and unsuitable for early screening. Furthermore, pathological diagnosis, determined by microscopic examination of tissue or cytology specimens acquired by invasive approaches, represents the gold standard for diagnosing lung cancer. Invasive methods are used to obtain lung tissue, including transbronchial or percutaneous fine needle biopsy (FNA), thoracoscopic biopsy and thoracotomy (8, 11, 12). FNA is the most commonly used for patients with peripheral lung nodules, but tumor lesions are usually small in size, difficult to obtain, and yield unsatisfactory results. In case of negative or ambiguous pathological results from tissue obtained by FNA, repeated biopsies or more invasive surgical procedures such as thoracoscopic wedge resection or thoracotomy are often indicated for definitive diagnosis. These invasive procedures can lead to serious complications, such as bleeding and pneumothorax, aggravate the economic burden of patients, delay the state of illness and even increase the risk of death (11, 12). Therefore, safer methods, which can provide a reliable basis for clinical diagnosis and treatment, are warranted for lung cancer screening and early diagnosis.

Following its popularity and widespread application, endobronchoscopy has been harnessed for screening, diagnosis and treatment of lung cancer because of its safety and efficiency (13–16). In recent years, several research groups have explored the diagnostic value of endobronchial optical coherence tomography (EB-OCT) in pulmonary nodules. Based on OCT, the pulmonary structural changes could be displayed in real-time and safely without invasive biopsy, yielding results highly consistent with pathological findings. In addition, EB-OCT combined with virtual bronchoscopy and electromagnetic localization techniques can guide transbronchial lung biopsy (TBLB) or transbronchial needle aspiration (TBNA) for pathological examination more accurately and in real-time. At the same time, EB-OCT can be used to localize and characterize the solitary pulmonary nodules which cannot be reached by routine bronchoscopy.

In recent years, researchers have made significant inroads in utilizing EB-OCT to diagnose lung cancer. We systematically reviewed EB-OCT publications published from January 1991 to November 2022, combining the overall composition, operating method and clinical application of EB-OCT. Moreover, the bottlenecks and difficulties in developing and popularizing EB-OCT for diagnosis and treatment during clinical practice are analyzed.

## 2 Optical coherence tomography

Optical coherence tomography (OCT) is a three-dimensional imaging technique based on the principle that near-infrared light waves have different refractive indices in different tissues. After computer analysis and processing, a two-dimensional real-time image of longitudinal and transverse sections is generated (17). The high resolution of 10-20μm, which is 50 times the resolution of HRCT images (18), afforded by OCT can display the local structure accurately and clearly, and the micro-structure images (19–21), which is highly consistent with the pathological results, can be observed in real-time in cases where no tissue biopsy is conducted.

Endobronchial optical coherence tomography is a new technique for observing endobronchial tissue structure *via* bronchoscopy equipped with an OCT probe in recent years. It is noninvasive, accurate, repeatable, radiation-free and easy to operate. OCT probes can enter the body through the fiberoptic bronchoscope working channel, and the structure of large, medium and small airways (up to 1-9 bronchi), the mucosal layer, submucosal layer, adventitia, alveoli, glands and cartilage of the bronchial wall can be displayed and accurately measured. The near-infrared (NIR) light band is harmless to patients with a penetration depth of 3mm into human tissues (22–24). EB-OCT examination can be conducted in conscious patients and avoid the risks from anesthesia, mechanical ventilation and other factors (25–27). Thus, the benefits of EB-OCT make it promising for diagnosing and treating lung cancer.

## 3 The components of EB-OCT

**Figure 1:**
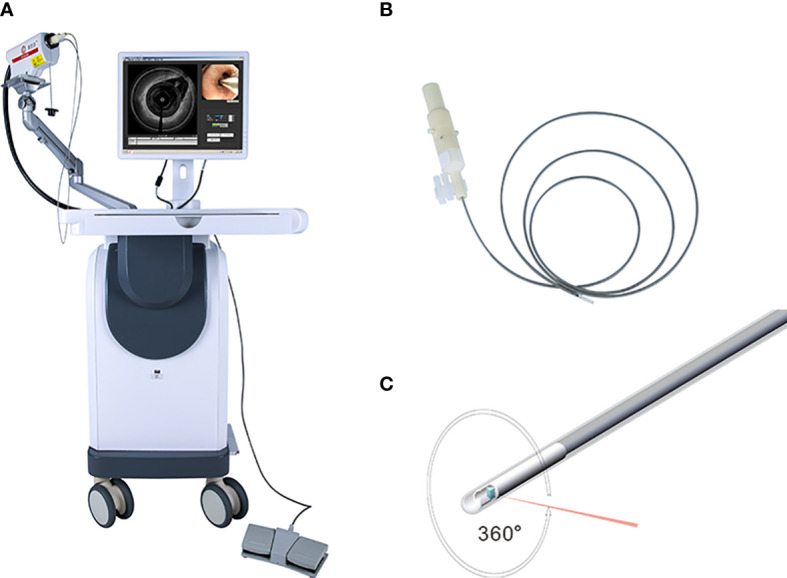
Showed the system of EB-OCT: **(A)** present the host of EB-OCT; the probe was displayed in **(B, C)**.

The EB-OCT operating system includes the main body of the tracheal mirror OCT system and probe (**Figure 1**). The parameters of the probe are as follows: Host function Laser wavelength: 1255-1355nm; detection depth: 0.3-1.5mm; scanning rate: ≥ 50kHz; optical sensitivity: ≥ 100db; scanning frame rate: ≥ 10 ten frames/second and inner diameter: 1.7mm ± 0.1mm and 2.5mm ± 0.1mm.

## 4 Operation process of EB-OCT

**Figure 2:**
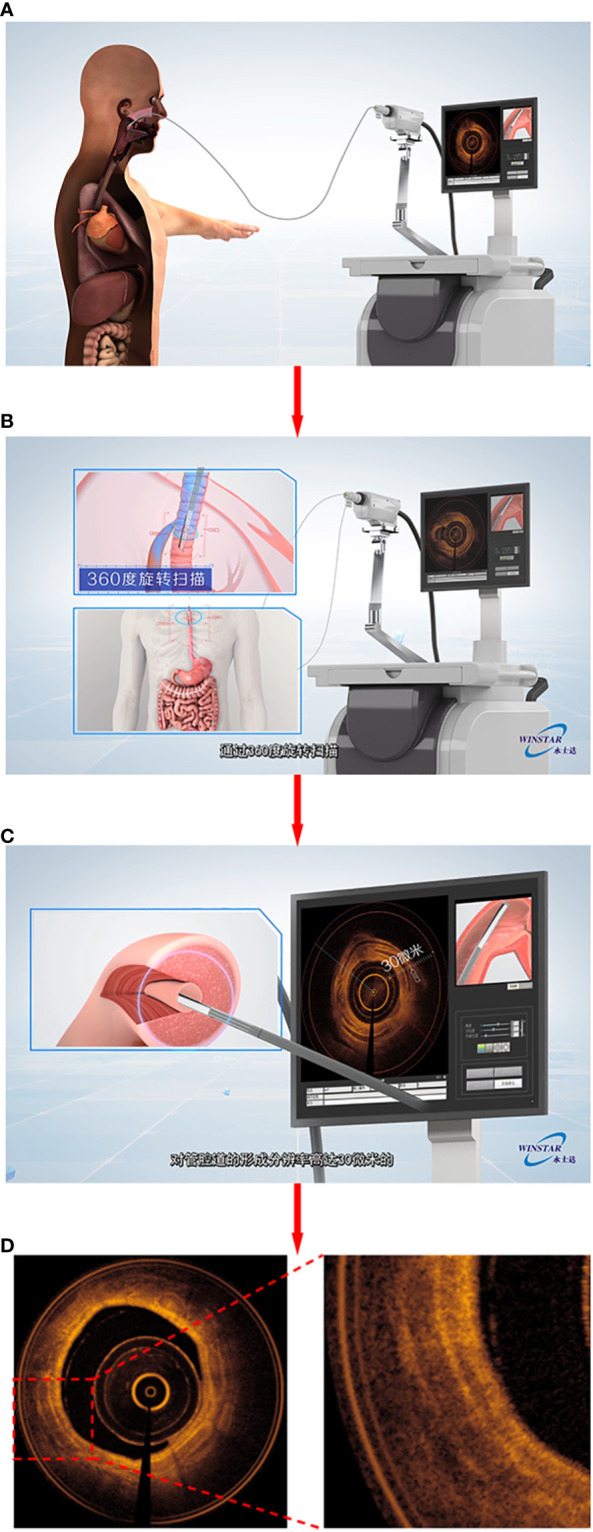
Demonstrated the process of EB-OCT examination. **(A)** showed that the probe enters the focus along the working channel of the bronchoscope; The probe scans 360 degrees in the cavity in **(B)**; **(C)** To form a cross-sectional picture of lumen tissue; **(D)** output airway OCT image similar to “pathological sections”.

All patients underwent HRCT one day before the bronchoscopy. Using the electromagnetic navigation system of the bronchoscope, HRCT reconstructed images could be converted into a three-dimensional bronchial tree image. Before the operation, the patient was given local anesthesia with 2% lidocaine solution atomization, and then the bronchoscope was inserted through the nose (Olympus B260f, Japan, with an outer diameter of 2.8mm and an inner diameter of 1.2mm). According to the planned path generated by the system, the bronchoscope was moved to the tertiary bronchi of the target lobe. The OCT probe (C7 dragonfly catheter) was then inserted into the working channel of the bronchoscope. After the opening of the tertiary bronchi was reached, the OCT probe was fixed at the opening, the patient was instructed to hold his/her breath after full inspiration and then the bronchoscope was pulled back to the segmental bronchus of the target segment. During the scanning process, the midpoint of single-segment bronchi was selected for measurement; the average lumen diameter, lumen area, airway wall area and other data were acquired for the segmental bronchi to the tertiary bronchi of the target lung segment, and automatic scanning and pullback was conducted. This procedure was repeated 3 to 5 times until three satisfactory images and data were obtained (**Figure 2**).

## 5 The applications of EB-OCT on respiratory diseases

### 5.1 Differential diagnosis of airway lesions

**Figure 3:**
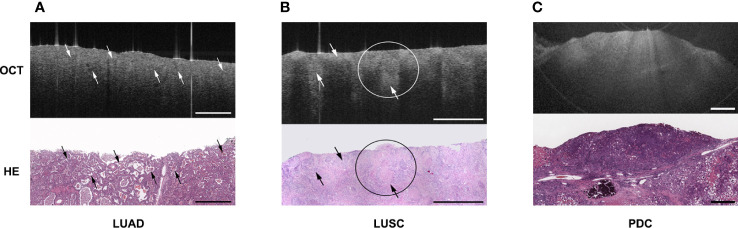
Showed the OCT images and corresponding H&E-stained histologic sections of different subtype of lung cancer: **(A)** lung adenocarcinoma (LUAD); **(B)** lung squamous cell carcinoma (LUSC); **(C)** poorly differentiated carcinoma (PDC). The picture was cited from Hariri LP, Mino-Kenudson M, Lanuti M, Miller AJ, Mark EJ, Suter MJ. Diagnosing lung carcinomas with optical coherence tomography. Ann Am Thorac Soc. 2015 Feb;12(2):193-201. doi: 10.1513/AnnalsATS.201408-370OC. PMID: 25562183; PMCID: PMC4342833.

Yang et al. (28) used EB-OCT to dynamically observe the growth of lung cancer cells *in vitro*. The morphology of the cell membrane under OCT was highly consistent with the image of conventional histopathology sections. These results suggest that OCT has great potential as a tool for rapid assessment of cell growth rate and morphology *in vitro* and may be used to evaluate the invasiveness of lung cancer cells. Tsuboi et al. (29)used OCT to observe the structure of normal bronchi, alveoli and central lung cancer. Compared with the uniform distribution of NIR light in normal bronchi, mucosa, submucosa and air-filled alveoli, uneven distribution of scattered light, loss of normal structure, and interval thickening between the epithelial surface and the cartilage were observed in the tumor area. Normal bronchial homogeneous mucosa, submucosa and alveoli are uniform cavity-containing structures, the tumor area is manifested as unevenly distributed high backscattering areas and resultant loss of the normal layer structure. Whiteman et al. (30)obtained OCT images that were highly consistent with the pathological tissue morphology of the bronchial cross sections of the lung after pneumonectomy and demonstrated that OCT findings were highly consistent with the histological findings in terms of structural contour and documented morphological changes involving inflammatory infiltrates, squamous metaplasia, and tumors using EB-OCT. Normal lung tissue showed clear layering and anatomic structure under OCT: epithelium, lamina propria, smooth muscle, mucinous gland and cartilage, with clear transition between each layer. The inflammatory infiltrates are poorly structured and the inflamed areas are more vascular, which makes them more absorbable. The squamous metaplastic epithelium manifested as a thicker area and can be distinguished from the normal monolayer epithelium. The location of the tumor showed the disappearance of the boundary of all levels of anatomical structures. In another clinical study (31), Michel et al. demonstrated the absence of normal bronchial structures on OCT scans in five patients with endobronchial tumors confirmed as lung cancer after further pathological biopsy at the same site. The structural changes during OCT imaging were highly consistent with pathological images, which suggested that OCT may replace traditional pathological biopsy and yield the same diagnostic effect without invasive tissue acquisition, and thus the concept of “optical biopsy” was first proposed. Mukherjee et al. (32)used OCT to image the lobectomy specimen’s tumor area and adjacent non-tumor area. The clinicians could judge the specific subtype of the tumor more accurately by the OCT images, and the results were consistent with the pathological examination obtained later. For pathological sections with ambiguous results, the OCT image characteristics could assist the reader in diagnosis. Jambeih et al. (33) selected 16 patients with intratracheal tumors and normal bronchi for scanning and image acquisition with EB-OCT, then biopsied the lesion. The normal bronchi could be visualized with normal epithelium, lamina propria, cartilage and other structures with clear boundaries on OCT images, while the lung cancer sites exhibited loss of normal structure/deformation or uneven dark lines on OCT images. The concept of “Optical fracture” was proposed, indicating that EB-OCT could effectively identify benign and malignant intratracheal lesions. OCT may be the safest diagnostic method for patients with contraindications to biopsy. It has been reported that the sensitivity and specificity of OCT for the detection of adenocarcinoma were 100% and 94%, respectively. Corresponding values for squamous-cell carcinoma were 80% and 100%, respectively. The overall sensitivity and specificity of OCT for lung cancer were 87.5% and 90.9%, respectively (34). These results suggest that OCT is effective in diagnosing airway adenocarcinoma and squamous-cell carcinoma. Hariri et al. (35) also demonstrated that OCT has good sensitivity and specificity for detecting adenocarcinoma, squamous cell carcinoma and poorly differentiated carcinoma and can be used as an adjunct to conventional pathological diagnosis when the biopsy specimens are insufficient (**Figure 3**).

### 5.2 Early lung cancer screening

**Figure 4:**
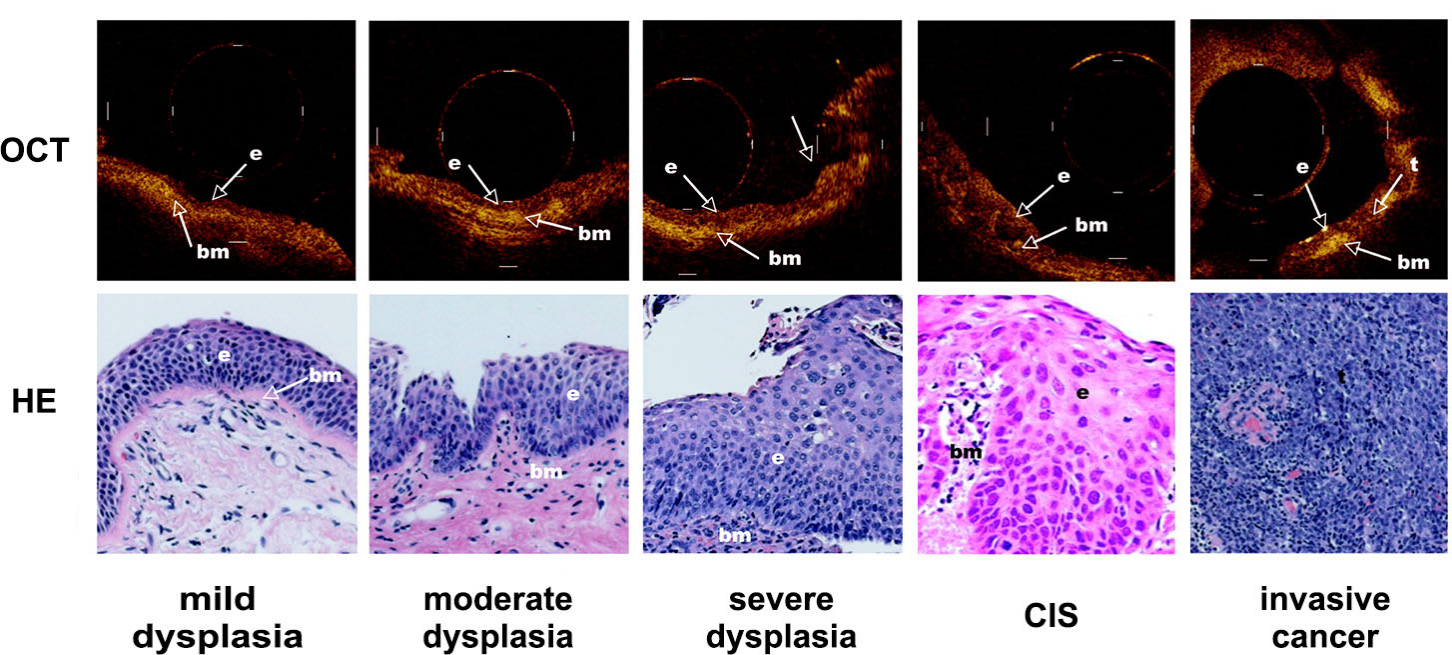
Demonstrated the dynamic process of lung cancer from middle dysplasia to invasive cancer. Representative OCT images of an area with mild dysplasia, moderate dysplasia, severe dysplasia, cancer in situ (CIS) and invasive cancer and corresponding H&E-stained histologic sections was showed. The picture was cited from Stephen Lam, Beau Standish, Corisande Baldwin, Annette McWilliams, Jean leRiche, Adi Gazdar, Alex I. Vitkin, Victor Yang, Norihiko Ikeda, Calum MacAulay; In vivo Optical Coherence Tomography Imaging of Preinvasive Bronchial Lesions. Clin Cancer Res 1 April 2008; 14 (7): 2006–2011. https://doi.org/10.1158/1078-0432.CCR-07-4418.

Precancerous lesions may not involve the airway mucosa during early-stage disease, and it is often difficult to identify the tumor before conducting a chest CT. Some studies (36) found a significant difference in permeability coefficient between normal and lung cancer tissue *in vitro*. These results suggest that OCT imaging combined with ultrasound may be a powerful tool for early diagnosis and monitoring changes in pulmonary microstructure. Lam et al. (20) found that in high-risk smokers, dysplasia and carcinoma *in situ* can be differentiated from hyperplasia or metaplasia, and invasive carcinoma can also be distinguished from carcinoma *in situ* by using OCT in combination with fluorescence bronchoscopy (**Figure 4**). On OCT images, dysplasia and carcinoma *in situ* were observed, with an increase in the number of epithelial cells and nuclei and a discontinuous basement membrane at the site of invasive carcinoma. Further quantitative OCT measurements showed an increase in epithelial thickness, consistent with histopathological grade and severity, suggesting that OCT may be useful for early screening and recognition of lung cancer (29).

### 5.3 Biopsy and localization of lung nodules and lymph nodes

Hariri et al. (37) described the characteristics of the OCT images of normal lung parenchyma and nodules *via* scanning normal lung parenchyma and nodules *in vitro*. Normal lung parenchyma was characterized by homogeneous signal voids in the alveoli and strong reflection area at the tissue-air interface, whereas the pulmonary nodules lacked these structures. The sensitivity and specificity of OCT for distinguishing nodules from normal lung parenchyma were 95.4% and 98.2%, respectively. Nandy et al. (38) combined EB-OCT with the Random Forest machine learning algorithm to evaluate the nature of peripheral pulmonary nodules. The results showed that the sensitivity, specificity and accuracy of OCT combined with the RF algorithm were 90.41%, 77.87% and 83.51%, respectively. Intra-airway navigation and biopsy of peripheral pulmonary nodules can be achieved using endobronchial ultrasound (EBUS). However, the currently available EBUS probe cannot identify large vessels, which may increase the risk of bleeding during tissue biopsy. EB-OCT also can be applied to facilitate lung biopsy. Zhang et al. (39) combined EBUS with OCT technology to identify the site of peripheral lung lesions with EBUS, generate OCT images, and finally perform a tissue biopsy of the abnormal area. A probe of 0.9 mm in diameter could identify peripheral pulmonary nodules before *in vivo* biopsy, improving the diagnostic rate of peripheral pulmonary nodules. In recent years, the combination of fluorescence technique and OCT has been extensively studied, which can accelerate the localization and identification of pulmonary nodules and be used for biopsy guidance of peripheral pulmonary nodules (40–42).

Significant progress has been made in exploring the nature of enlarged lymph nodes in the lungs. Robert et al. (21) performed OCT scanning *in vitro* on surgically resected lymph nodes; the OCT images of the resected enlarged lymph nodes were highly consistent with the histopathology images. Sampling error and higher false negative rates were observed during the sampling of lymph nodes by EBUS-TBNA. To improve the diagnosis of EBUS-TBNA, Shostak et al. (43) designed a novel biopsy needle with an OCT probe: normal lymph nodes showed homogeneous areas with moderate signal intensity and minimal microstructure, while malignant lymph nodes with cancer metastasis showed irregular dense nest-shaped signals. It has been suggested that OCT can describe and compare the morphologic features of benign and metastatic lymph nodes in the chest and identify the optimal puncture site for EBUS-TBNA to improve diagnostic accuracy.

The positivity rate of biopsy specimens has been a concern during clinical practice. The insufficient quantity and depth of specimens may reduce the diagnostic value of biopsy specimens and make it challenging to differentiate lesions from inflammation, fibrosis and calcification. Hariri et al. (44) used OCT to identify the degree of pulmonary fibrosis *via* tissue refractive index and collagen staining. The results showed that OCT could accurately classify the areas with fibrosis > 20% and those with ≤20% fibrosis. Nandy et al. (38) consistently used OCT to distinguish tumor, fibrotic and normal lung tissues according to the different characteristics of birefringence and depolarization uniformity (Dopu) of collagen in different tissues. The above parameters exhibited a good correlation with the pathological findings, with good specificity and sensitivity rates. This finding suggests that OCT can accurately identify fibrosis and differentiate low-fibrotic tumor areas, which may be used to assist in biopsy sampling of lung nodules or for rapid evaluation of surgical specimens. It plays a significant role in guiding the tissue sampling and surgical resection range during *in vivo* surgery. Kuo et al. (45) designed an integrated core needle biopsy system that combines OCT with a biopsy needle for adjuvant tissue biopsy, enabling real-time three-dimensional OCT imaging before and after tissue biopsy. Accurate identification of the biopsy area and timely evaluation of the quality of the biopsy tissue are expected to improve the positive rate of the biopsy and increase the validity of the biopsy results.

### 5.4 Palliative treatment of lung cancer

Airway stent implantation is a common palliative approach in cases of advanced lung cancer, which can significantly improve symptoms such as dyspnea and chest tightness. However, it can be challenging to measure the diameter of large airways and choose the appropriate diameter stent. Williamson et al. (46) attempted to use OCT to assess airway diameter in real time and guide stent implantation with good results. Other studies (47, 48) have used EB-OCT to accurately measure large airway diameter and luminal area in real-time during bronchoscopy, which can guide the selection of airway stent diameter. In addition, OCT can image airway obstruction and areas outside the field of vision of the bronchoscope to determine the extent of tumor obstruction. Indeed, OCT has more clinical advantages than LDCT in evaluating the degree of airway stenosis.

Patients with advanced cancer often need endotracheal intubation and laser ablation of the intratracheal tumor. Evaluating the degree of airway injury helps determine the treatment approach. OCT can display the structure of the larynx and airway wall, dynamically observe the inflammation of airway mucosa and submucosa, scar fibrosis and adjuvant therapy (49, 50).

## 6 Challenges

Although OCT has high sensitivity and specificity in identifying adenocarcinoma, squamous cell carcinoma and poorly differentiated carcinoma, these results are not enough to support the status of OCT as the “gold standard” instead of histopathological biopsy. Indeed, there are still some false-positive and false-negative cases of adenocarcinoma and squamous cell carcinoma; a study reported that 56% of poorly differentiated adenocarcinomas were diagnosed as adenocarcinomas by OCT (35), suggesting that final histopathological validation is still required. The accuracy of OCT diagnosis is related to the ability of readers to recognize images. The diagnostic rate of lung cancer can be significantly improved by pathologists with OCT experience, suggesting that the training methods and training time for novice readers have an important impact on the results. A widely recognized OCT reading training process is warranted to standardize the training time and process, with strict adherence to the standards of OCT image recognition to improve the diagnostic accuracy of non-OCT expert readers.

## 7 Conclusion

Based on its high safety and efficacy, EB-OCT provides an alternative for clinical diagnosis and treatment. It can accurately distinguish different lesions such as lung parenchyma, lung nodule, lung squamous cell carcinoma, lung adenocarcinoma, and carcinoma in situ, consistent with the histopathological results. OCT examination is relatively non-invasive, with high accuracy, repeatability, and short operation time, and has a relatively simple interpretation process suitable for clinical implementation. OCT can image and evaluate the lung and peripheral lesions, assist in transbronchial needle aspiration biopsy, and evaluate the quality of samples in real-time. EB-OCT also plays a role in the palliative treatment of advanced lung cancer. However, the currently available evidence is still insufficient to support the replacement of OCT as the “gold standard” instead of histopathology. In the future, an OCT map recognition training process should be established to improve the diagnostic accuracy of non-OCT expert readers with a standardized training process strictly abiding by the OCT map recognition standards.

## Author contributions

Conception and design: HL, and YY. Administrative support: HL, JJ, LC, and YY. Provision of study materials or patients: HL, JJ, LC, JF, JL and YY. Collection and assembly of data: HL, JJ, LC, JF, and JL. Data analysis and interpretation: All authors. Manuscript writing: All authors. All authors contributed to the article and approved the submitted version.
